# Cytosolic galectin-7 impairs p53 functions and induces chemoresistance in breast cancer cells

**DOI:** 10.1186/1471-2407-14-801

**Published:** 2014-11-03

**Authors:** Andrée-Anne Grosset, Marilyne Labrie, Donald Gagné, Maria-Claudia Vladoiu, Louis Gaboury, Nicolas Doucet, Yves St-Pierre

**Affiliations:** INRS-Institut Armand-Frappier, 531 Blv. des Prairies, Laval, Quebec H7V 1B7 Canada; IRIC | Université de Montréal, 2950 Chemin de Polytechnique, Montreal, Quebec H3T 1J4 Canada

**Keywords:** Galectin-7, Localization, Apoptosis, p53, Breast cancer

## Abstract

**Background:**

Resistance to apoptosis induced by anti-cancer drugs is a major obstacle for the treatment of aggressive forms of breast cancer. Galectin-7 (gal-7) was recently shown to be specifically expressed in basal-like but not in luminal subtypes of human breast cancer.

**Methods:**

We generated a mutant form of gal-7 (R74S). Arginine 74 is the structural equivalent of arginine 186 found in human galectin-3. Mutation R186S was previously shown to abolish the biological function of galectin-3.

**Results:**

Mutation of arginine 74 induced only limited and local changes to the gal-7 fold. Recombinant forms of R74S and wtgal-7 were also equally effective at forming dimers in solution. Analysis of the thermodynamic parameters by isothermal titration calorimetry (ITC) indicated, however, that binding of lactose to gal-7 was inhibited by the R74S mutation. Using confocal microscopy and electron microscopy, we confirmed the expression of gal-7 in the cytosolic and nuclear compartments of breast cancer cells and the ability of gal-7 to translocate to mitochondria. The mutation at position 74, however, greatly reduced the expression of gal-7 in the nuclear and mitochondrial compartments. Interestingly, cells expressing mutated gal-7 were equally if not even more resistant to drug-induced apoptosis when compared to cells expressing wtgal-7. We also found that both wtgal-7 and R74S inhibited dox-induced PARP-1 cleavage and p53 protein expression. The inhibition of p53 correlated with a decrease in p21 protein expression and *CDKN1A* mRNA. Furthermore, analysis of nuclear and cytoplasmic fractions showed that both wild type and R74S mutant gal-7 inhibited p53 nuclear translocation, possibly by increasing degradation of cytosolic p53.

**Conclusions:**

These findings pose a challenge to the paradigm that has guided the design of galectin-specific inhibitors for the treatment of cancer. This study suggests that targeting CRD-independent cytosolic gal-7 in breast cancer cells may be a valuable strategy for the treatment of this disease. Our study will thus complement efforts towards improving selectivity of targeted anticancer agents.

**Electronic supplementary material:**

The online version of this article (doi:10.1186/1471-2407-14-801) contains supplementary material, which is available to authorized users.

## Background

Members of the galectin family are characterized by their ability to bind β-galactosides via a highly conserved carbohydrate recognition domain (CRD). They play an important role in several physiological processes, including embryonic development, intercellular adhesion, host-pathogen interactions, cell migration, and immune response [1]. They are normally classified according to their structural organization. Galectins containing only one CRD are called prototype and include galectins 1, 2, 5, 7, 10, 11, 13, 14 and 15. Those with two distinct CRDs in tandem connected by a linker region (tandem-repeat type) are galectins 4, 6, 8, 9 and 12. Galectin-3 is the only member of the third group and is a chimera-type protein with one CRD connected to an unusual non-lectin domain rich in proline and glycine.

Historically, galectins have been known as small extracellular soluble that bind cell surface glycans, helping organizing membrane domains and regulating the signaling threshold and the receptor residency time [2]. Galectins, however, exhibits a wide range of subcellular localizations, being found in both intracellular and extracellular compartments. Intracellularly, they have been reported to be exclusively/predominantly cytosolic, nuclear, mitochondrial, or distributed between the distinct subcellular compartments. Finally, even within a specific organelle, they appear to be distributed diffusely or to form aggregates or punctate structures. Such wide subcellular distribution significantly complicate galectin-targeted anticancer therapy since the pro- and the anti-tumoral functions of galectins differ according their subcellular localization [3].

Galectin-7 (gal-7) is a prototype galectin that forms homodimers [4]. Gal-7 is preferentially expressed in stratified epithelia, including epidermis, cornea, oral cavity, esophagus and rectal epithelium [5]. It is also expressed in mammary myoepithelial cells in tissues of normal individuals [6]. Its level of expression, however, is significantly altered in various types of cancer [7]. For example, gal-7 is expressed at higher levels in aggressive molecular subtypes of breast carcinoma, most notably in basal-like breast cancer with an ER/PR/HER-2 negative status [6]. Exogenous expression of gal-7 in breast cancer cell lines that express low or undetectable levels of gal-7 resulted in an increased metastatic behavior to the lung and bone and larger osteolytic lesions. Such pro-tumoral function of gal-7 has been largely attributed to its ability to protect cancer cells from pro-apoptotic signals [6, 8]. Like other galectins, however, gal-7 is preferentially expressed intracellularly, most notably in cytosolic, nuclear and mitochondrial compartments [9–12]. Whether the resistance of breast cancer cells to apoptosis is dependent on the intracellular localization of gal-7 remains unknown. In the present work, we have addressed this question by generating a mutant form of gal-7 (R74S) with altered subcellular localization and tested its ability to mediate resistance of breast cancer cells to drug-induced cell death.

## Methods

### Tissue microarrays and immunohistochemistry

Representative specimens from our previous TMA analysis were immunostained for gal-7 using the Discovery XT automated immunostainer (Ventana Medical Systems, Tucson, AZ) [6]. Deparaffinized sections were incubated in Cell Conditioning 1 (pH 8.0) for antigen retrieval and then stained for 60 min with the anti-human gal-7 polyclonal antibody (R&D Systems, Minneapolis, MN) using a 1:150 dilution. The slides were counterstained with hematoxylin and bicarbonate. Each section was scanned at a high resolution using the Nanozoomer Digital Pathology System (Hamamatsu, Bridgewater, NJ). The study was approved by the research ethics committee of the research center at the Centre Hospitalier de l’Université de Montréal (approval No. SL 05.019).

### Cell lines and reagents

The MCF-7 and MDA-MB-468 cell lines were provided by Dr. Peter Siegel (Rosalind and Morris Goodman Cancer Research Centre, McGill University, Montreal, QC, Canada) and maintained in Dulbecco’s Modified Eagle’s Medium (DMEM) supplemented with 10% (v/v) FBS, 2 mM L-glutamine, 10 mM HEPES buffer and 1 mM sodium pyruvate. SKBR3 cells, obtained from Dr. Sylvie Mader (Institute for Research in Immunology and Cancer, University of Montreal, Montreal, QC, Canada), were grown in McCoy’s 5A Medium supplemented with 10% (v/v) FBS, 2 mM L-glutamine and 10 mM HEPES buffer at 37°C in a humidified atmosphere containing 5% CO_2_. MCF10A and MCF12A protein extracts were provided by Dr. Isabelle Plante (INRS-Institut Armand-Frappier, Laval, QC, Canada). All cell culture products were purchased from Life Technologies (Burlington, ON, Canada). Cobalt chloride and lactose were purchased from Fisher Scientific (Ottawa, ON, Canada). MG-132 was from Cayman Chemical (Ann Arbor, MI). All other reagents were purchased from Sigma-Aldrich (St. Louis, MO), unless otherwise indicated.

### Generation of stable transfectants expressing gal-7 and gal-7 R74S

To obtain stable MCF-7 breast carcinoma transfectants expressing gal-7, the cDNA encoding the human gal-7 (provided by Dr. Thierry Magnaldo) was cloned in srα eukaryotic expression vector (kind gift of Dr. François Denis) using *Spe*I and *Bam*HI restriction enzymes. The replacement of arginine 74 to serine (R74S) was introduced by oligo-directed site-specific mutagenesis using the forward (5′-GGC CGC GAG GAG TCC GGG CCG GGC GTT CCT- 3′) and reverse (5′ –GGC CGC GAG GAG TCC GGG CCG GGC GTT CCT- 3′) primers. Controls were generated using MCF-7 breast carcinoma cells transfected with the empty srα vector. Transfection was carried out using Lipofectamine 2000 according to the manufacturer’s instructions (Life Technologies). After 48 h of culture, transfected cells were allowed to grow in complete medium containing 1 μg/ml of puromycin. Individual colonies were expanded and gal-7 expression was monitored by Western blot analysis. All experiments were conducted with at least two independent clones expressing either wild type or mutant gal-7.

### RNA isolation ant RT-PCR

Total cellular RNA was isolated from cells using the TRIzol reagent (Life Technologies) according to the manufacturer’s instructions. First-strand cDNA was prepared from 2 μg of cellular RNA in a total reaction volume of 20 μL using the reverse transcriptase Omniscript (QIAGEN, Mississauga, ON, Canada). After reverse transcription, human *p53* (gene ID 7157, sense primer: 5′- CCA GCC AAA GAA GAA ACC A -3′ and antisense primer: 5′- TAT GGC GGG AGG TAG ACT GA -3′), human *p21* (gene ID 1026, sense primer: 5′- CTG GAG ACT CTC AGG GTC GAA -3′ and antisense primer: 5′- GGA TTA GGG CTT CCT CTT GGA -3′) and *GAPDH* (gene ID 2597, sense primer: 5′- CGG AGT CAA CGG ATT TGG TCG TAT-3′ and antisense primer: 5′-CAG AAG TGG TGG TAC CTC TTC CGA -3′) cDNAs were amplified using the following conditions: 94°C for 3 min, followed by 25 to 35 cycles of the following: 94°C for 40 seconds, 60°C for 40 seconds, and 72°C for 40 seconds, followed by a final extension step at 72°C for 10 min. PCR was performed in a thermal cycler (Eppendorf, Mississauga, ON, Canada). The amplified products were analyzed by electrophoresis using 1.5% agarose gels and SYBR Safe (Life Technologies) staining and UV illumination.

### Co-immunoprecipitation

MCF-7 stable transfectants expressing exogenous gal-7 and R74S mutant and MCF10A were transfected with vectors encoding wild type p53 (Origene, Burlington, MA). After 24 hrs, the cells were lysed in immunoprecipitation (IP) buffer containing 2% (v/v) CHAPS, 50 mM Tris pH 7.5, 150 mM NaCl, 0.1 mM EDTA and protease inhibitors (Roche, Laval, QC, Canada). Equal amounts of whole cell protein extracts were used for each IP. Rabbit anti-p53 antibody (FL393; Santa Cruz Biotechnology, Santa Cruz, CA) or IgG control antibody (2 μg) were incubated 10 min at room temperature with Dynabeads Protein G (Life Technologies). The Dynabeads-antibody complex was incubated with proteins overnight at 4°C. After several washes in IP buffer, the protein complexes were resuspended in Laemmli loading buffer. Immunoprecipitated proteins were separated on a 15% SDS-PAGE gel and analyzed by Western blotting using anti-gal-7 and anti-p53 as described below.

### Western blot analysis

Whole cell extracts were suspended using RIPA lysis buffer (Thermo Fisher Scientific, Rockford, IL) and protease inhibitors (Roche). Mitochondria and nuclear proteins were extracted using a kit (Thermo Fisher Scientific; Sigma-Aldrich) following the manufacturer’s instructions. Protein concentrations were measured using a protein assay reagent (Bio-Rad Laboratories, Mississauga, ON, Canada). Equal amounts of proteins were separated on SDS-PAGE and transferred onto nitrocellulose membranes (Bio-Rad Laboratories). The membranes were first blocked with 5% (v/v) milk in PBS/0.05% Tween 20 for 1 h and subsequently blotted overnight at 4°C with primary antibodies: goat anti-human gal-7 polyclonal antibody (1:1000; R&D Systems, Minneapolis, MN), rabbit anti-p53 (FL393; 1:1000; Santa Cruz Biotechnology), rabbit anti-p21 (1:1000; Cell Signaling Technology, Danvers, MA), rabbit anti-poly (ADP-ribose) polymerase (PARP)-1 (p25) monoclonal antibody (1:10000; Epitomics, Burlingame, CA), rabbit anti-COX IV polyclonal antibody (1:1000; Cell Signaling Technology), mouse anti-lamin A/C monoclonal antibody (1:1000; Cell Signaling Technology), rabbit anti-β-tubulin monoclonal antibody (1:10000; Cell Signaling Technology), and mouse anti-β-actin monoclonal antibody (1:20000; Sigma-Aldrich). Secondary antibodies consisted of horseradish peroxidase conjugated donkey anti-goat (R&D Systems), anti-rabbit or anti-mouse (GE Healthcare, Buckinghamshire, England). Detection was performed by the enhanced chemiluminescence method (GE Healthcare).

### Immuno-electron microscopy

Cells were fixed in a 0.1% (v/v) gluteraldehyde and 4% (v/v) paraformaldehyde solution and embedded in the low viscosity embedding Spurr media. Ultrathin sections were cut, placed on nickel grids and incubated in sodium metaperiodate. Samples were blocked in 1% (v/v) BSA for 5 min, incubated 60 min in a goat anti-human gal-7 polyclonal antibody (1:150) and 60 min in a rabbit anti-goat 10 nm gold-conjugated secondary antibody (1:20, Electron Microscopy Sciences, Hatfield, PA). Each section were counterstained with uranyle acetate and lead citrate and visualized using a Hitachi 7100 transmission electron microscope.

### Apoptosis detection by flow cytometry

The percentage of apoptotic cells was measured by two-color flow cytometry using Alexa Fluor 488 annexin V conjugate (Life Technologies) and propidium iodide (PI). Briefly, 1.75 × 10^5^ cells were treated with 150 μM cobalt chloride overnight at 37°C without serum. Cells were then harvested, stained and analyzed by flow cytometry using a FacsCalibur (BD Biosciences, San Jose, CA).

### Production of recombinant gal-7

Each of the DNA fragments coding for gal-7 and R74S was cloned into pET-22b(+) using *Nde*I and *Hin*dIII restriction enzymes. Recombinant proteins were expressed in *E. coli* BL21(DE3) at 37°C following addition of 1 mM IPTG at an OD_600 nm_ = 0.6-0.7 and an incubation of 4 h. Bacterial pellets were resuspended in lysis buffer (0.7 mg/mL lysozyme, 10 mM Tris pH 8, 100 mM NaCl, 1 mM EDTA, 1 mM DTT and protease inhibitor cocktail), incubated for 1 h at 37°C and centrifuged for 30 min at 15 000 *g* (4°C). The supernatant was then filtered and applied to a lactose-agarose column and the protein was eluted in one mL fractions with 150 mM lactose. Fractions were analyzed by SDS-PAGE. Gal-7 and R74S were dialyzed against 20 mM potassium phosphate at pH 7.2 for all subsequent characterization experiments. ^15^N-labeled samples were prepared by growing *E. coli* BL21(DE3) in M9 minimal medium as previously described [13].

### Solution NMR experiments

Proteins were at a concentration of 535 μM for gal-7 and 200 μM for gal-7 R74S. A 10% (v/v) D_2_O solution was added to the protein samples for NMR spin-lock purposes. Protein concentration was determined by UV–vis spectrophotometry using an extinction coefficient of 8030 M^-1^ cm^-1^ at 280 nm [14]. ^1^H-^15^N HSQC spectra were acquired at 800 MHz on a Varian (Agilent) NMR spectrometer equipped with a triple resonance probe and pulsed-field gradients. All spectra were acquired at 310 K as calibrated with a standard methanol sample. The ^1^H-^15^N HSQC experiments were conducted with 256 *t*_1_ and 8192 *t*_2_ points with proton and nitrogen spectral widths of 3000 and 8000 Hz, respectively. Spectra were processed using NMRPipe [15] and further analysed using Sparky [16]. The ^1^H-^15^N composite chemical shift differences (Δδ) were calculated between wild-type and mutant enzymes according to the following equation [17]: Δδ (ppm) = [(Δδ^2^_HN_ + Δδ^2^_N_/25)/2]^½^. Only chemical shift variations showing Δδ >0.02 ppm were considered significant.

### Isothermal titration calorimetry (ITC)

Lactose was reconstituted in a 20 mM potassium phosphate buffer at pH 7.2. Gal-7 and R74S were dialyzed in the same buffer after purification. All experiments were performed in a Nano ITC microcalorimeter (TA Instruments, New Castle, DE) at 25°C with a stirring rate of 250 rpm. Pre-equilibrated solutions of 200 μM protein and 6 mM ligand were used for each assay. A control experiment was performed by titrating lactose into protein-free buffer. Each experiment consisted of 20 injections of 2 μL ligand into protein, with an interval of 130 seconds between injections. All experiments were performed at least in triplicate. Data was analyzed and fitted using the NanoAnalyze software v2.3.6 (TA Instruments).

### FITC conjugation and gal-7 binding assay

Briefly, 10 μl of a 2 mg/ml fluorescein isothiocyanate (FITC)/DMSO solution was added to 300 μl of 1.7 μg/μl recombinant wtgal-7 or R74S in a 0.1 M NaHCO3 pH 9.2 solution and incubated for 2 hrs at room temperature on a roller. FITC-conjugated wtgal-7 or R74S was then purified using a PD-10 sepharose column (GE healthcare) and eluted with PBS containing 0.01% [v/v] sodium azide (PBA). To measure FITC-wtgal-7 or R74S binding to cell surface, 2.5 × 10^5^ cells were incubated for 30 min with the indicated concentrations. Cells were washed 2 times with PBA and resuspended in 500 μl PBA. Samples were analyzed by FACSCalibur (BD Biosciences).

## Results

### Subcellular localization of gal-7 in human breast cancer cells

**Figure 1:**
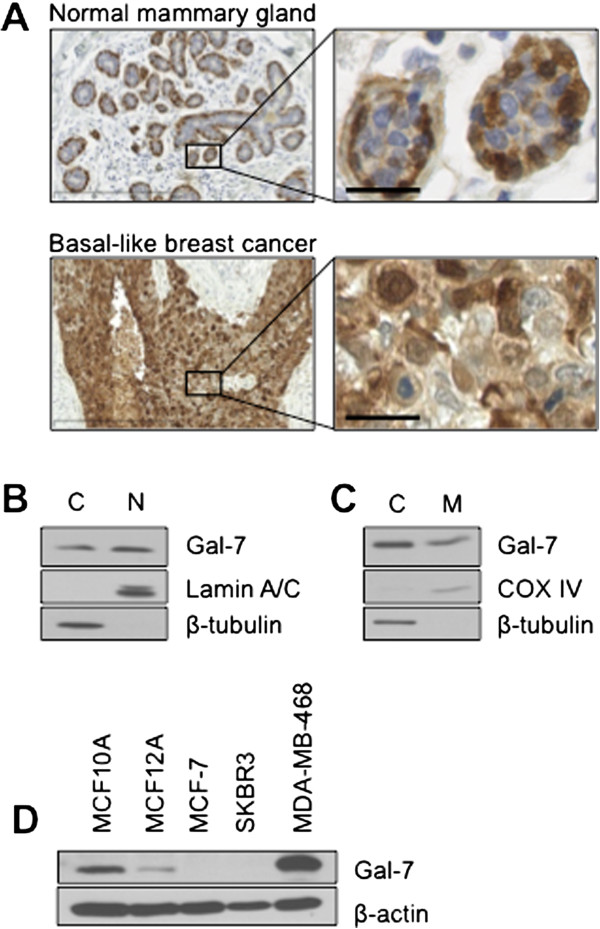
**Expression of gal-7 in human mammary cancer tissues and cell lines. (A)** Typical expression patterns of gal-7 in normal mammary tissues (myoepithelial cells) and in basal-like tumors collected from patients. Detailed immunohistochemical analysis in different types of breast cancer patients have been reported previously [6]. Scale bars, 300 μm (left) and 25 μm (right). **(B)** Cytosolic and nuclear fractions were purified from basal-like breast cancer cells MDA-MB-468. Expression of endogenous gal-7 was measured in both fractions by Western blotting. β-tubulin and lamin A/C are shown as positive cytoplasmic and nuclear expression controls, respectively. **(C)** Similar analysis showing expression of gal-7 in purified mitochondrial fractions. β-tubulin and COX IV are shown as positive cytoplasmic and mitochondrial expression controls, respectively. **(D)** Gal-7 in human breast cancer cell lines. Expression was measured by Western blot analysis from whole cell lysates. β-actin is shown as a positive expression control.

Analysis of gal-7 expression in normal mammary epithelium shows positive gal-7 staining in both the nuclear and cytoplasmic compartments of myoepithelial cells (Figure 1A). A similar pattern of expression was observed in tissue sections obtained from patients with breast cancer, most notably in sections from patients with basal-like breast cancer, where gal-7 is preferentially expressed [6]. The expression of gal-7 in cytosolic and nuclear compartments was also confirmed by western blot analysis of subcellular fractions isolated from MDA-MB-468 cells, which constitutively express gal-7 (Figure 1B-D). We also confirmed the ability of gal-7 to translocate to mitochondria in breast cancer cells, as recently observed in human colon carcinoma cells [11].

### Generation of the R74S mutant

**Figure 2:**
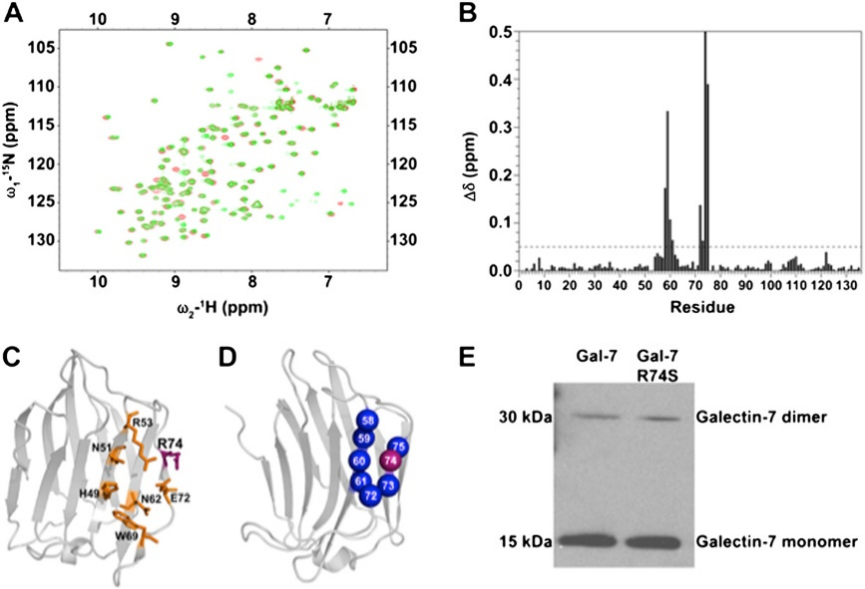
**Structural analysis of wild-type gal-7 and the R74S mutant. (A)** Superimposed ^1^H-^15^N HSQC spectra of wild-type (green) and R74S (red) gal-7 at 310 K and 800 MHz. **(B)** ^1^H-^15^N chemical shift differences Δδ (ppm) caused by the R74S mutation mapped on the primary sequence of gal-7. The ^1^H-^15^N weighted average composite chemical shift differences (Δδ) were calculated between WT and variant R74S according to the following equation [17]: Δδ (ppm) = [(Δδ^2^_HN_ + Δδ^2^_N_/25)/2]^½^. **(C)** Three-dimensional structure of gal-7 showing the general β-sheet topology of the carbohydrate recognition domain (CRD) and the position of the carbohydrate binding site, as delineated by residues H49, N51, R53, N62, W69, E72 and R74. **(D)** Mapping of ^1^H-^15^N chemical shift variations (Δδ) between wtgal-7 and variant R74S on the 3D structure of gal-7 [PDB: 3ZXF]. Residues with chemical shift variations Δδ >0.05 ppm are plotted on the structure of gal-7 (in blue). The position of the R74 residue is shown in purple. **(E)** Immunoblots showing soluble monomeric and dimeric forms of recombinant gal-7 in a native gel.

Site-directed mutagenesis was used to generate mutants of gal-7. A special attention was paid to arginine 74, the structural equivalent of arginine 186 in human galectin-3. Mutation R186S was previously shown to abolish the biological function of galectin-3 [18]. The replacement of arginine 74 to serine (R74S) was thus introduced in the human gal-7 gene by oligo-directed site-specific mutagenesis. To verify the integrity and structural perturbations caused by the R74S mutation, we used solution NMR spectroscopy, which provides a fast and highly sensitive assessment of structural perturbations caused by point mutations in proteins. For this purpose, the wild-type gal-7 (wtgal-7) and variant R74S were isotopically labeled, overexpressed in *E. coli* BL21(DE3) and purified to homogeneity. Their two-dimensional heteronuclear single quantum coherence spectra (^1^H-^15^N HSQC) were then acquired and overlaid (Figure 2A). Our ^1^H-^15^N HSQC spectral analysis showed that the R74S mutation induced only limited and local changes to the gal-7 fold (Figure 2A-D). Recombinant forms of R74S and wtgal-7 were also equally effective at forming dimers in solution (Figure 2E). Analysis of the thermodynamic parameters of the proteins by isothermal titration calorimetry (ITC) indicated, however, that binding of lactose to gal-7 was partially inhibited by the R74S mutation, with a *Κ*_d_ value of 720 μM for the R74S variant relative to 378 μM for the wild-type protein. A typical titration profile is shown in Additional file 1: Figure S1. This finding was corroborated by our flow cytometric analysis showing that binding of recombinant R74S to glycan receptors on the surface of Jurkat T cells was significantly lower than that observed with the wild-type protein (Additional file 2: Figure S2).

### Functional characterization of the R74S mutant

**Figure 3:**
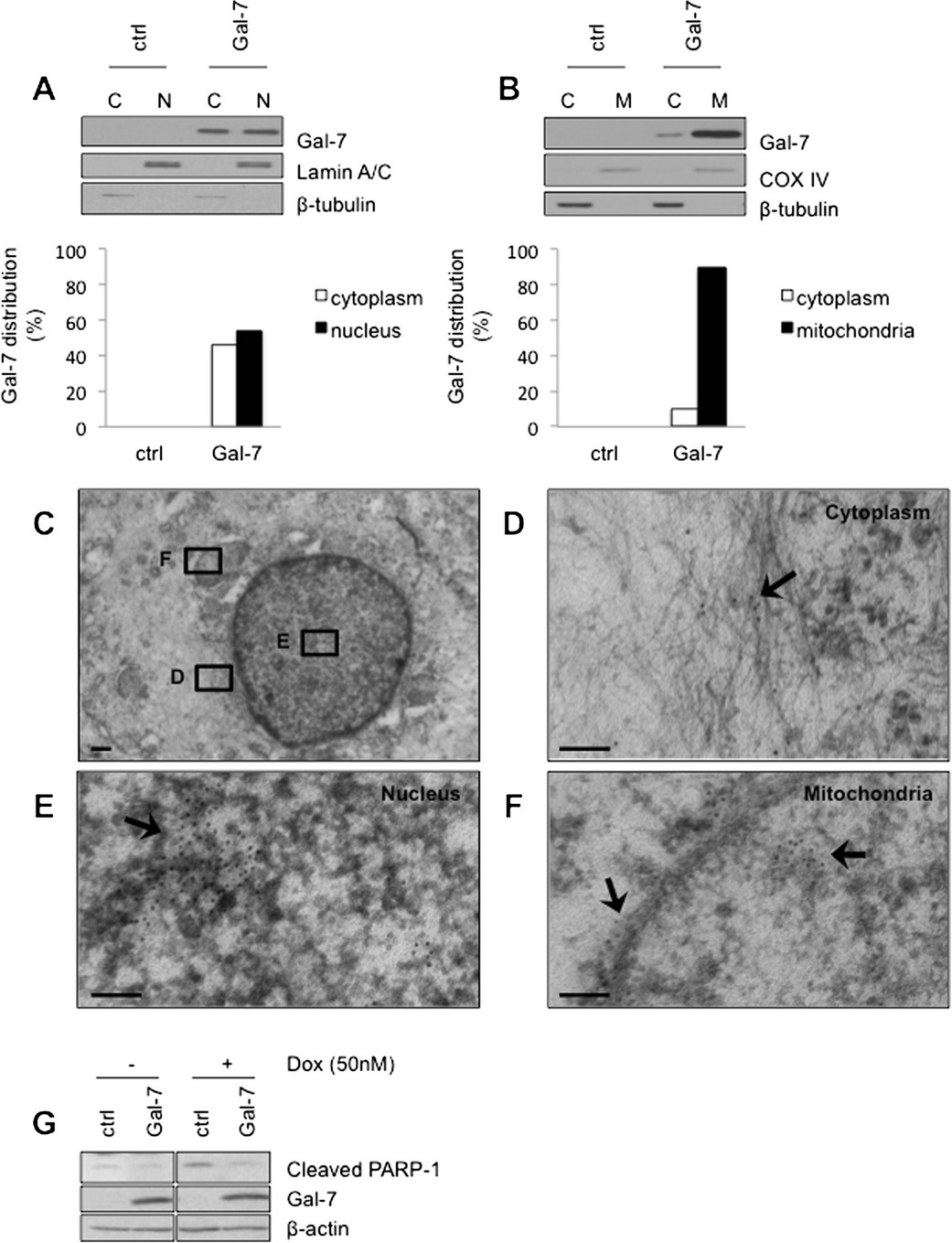
**Subcellular distribution of gal-7 in MCF-7 cells. (A)** Cytosolic and nuclear fractions were purified from control (srα) cells and MCF-7 cells expressing gal-7. Expression of gal-7 was measured in both fractions by Western blotting. β-tubulin and lamin A/C are shown as positive cytoplasmic and nuclear expression controls, respectively. **(B)** Similar analysis showing expression of gal-7 in purified mitochondrial fractions. β-tubulin and COX IV are shown as positive cytoplasmic and mitochondrial expression controls, respectively. **(C-F)** Subcellular localization of gal-7 in MCF-7 as measured by electron microscopy. Scale bars, 500 nm **(C)** and 100 nm **(D-F)**. **(G)** Effect of gal-7 on induction of apoptosis by doxorubicin. Stable transfectants of MCF-7 cells expressing gal-7 were treated with 50 nM doxorubicin for 72 hrs at 37°C. Cells were then harvested and PARP-1 cleavage levels were assayed by Western blotting.

**Figure 4:**
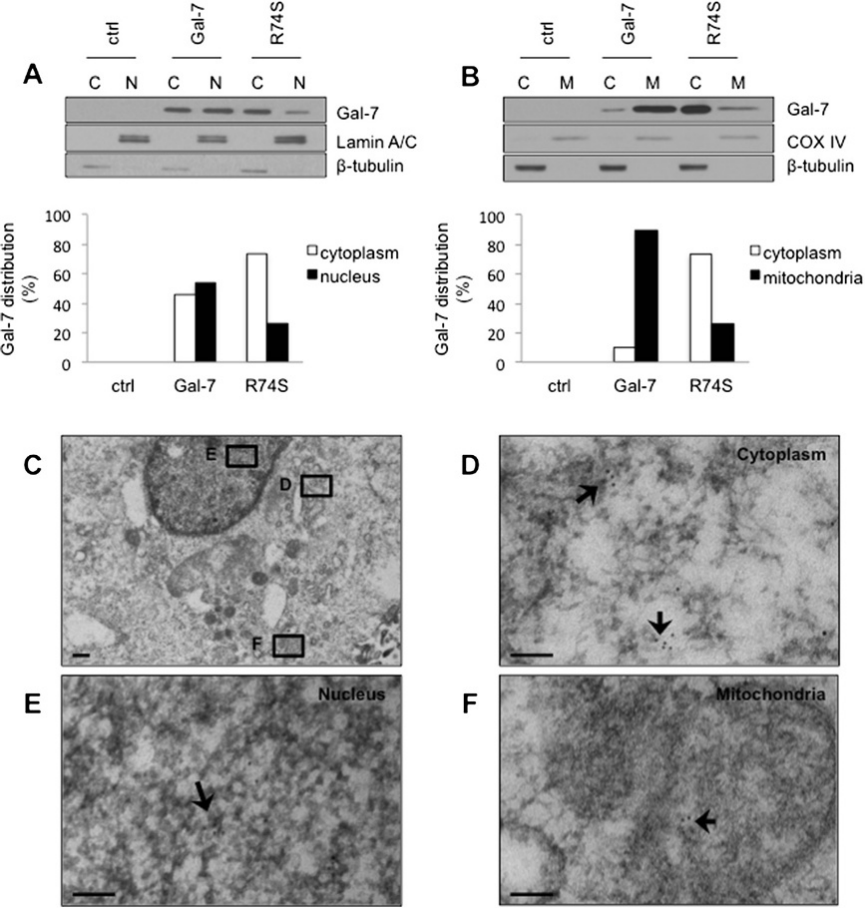
**Subcellular distribution of the R74S mutant.** The effect of the R74S mutation on the **(A)** nuclear and **(B)** mitochondrial localization of gal-7, as measured by Western blot analysis of subcellular fractions. Membrane blots were incubated with anti-gal-7, anti-lamin A/C, anti-COX-IV and anti-β-tubulin. Lamin A/C, COX-IV and β-tubulin were used as nuclear, mitochondrial and cytosolic markers, respectively. **(C)** Whole MCF-7 cell and distribution of galectin-7 R74S mutant in **(D)** cytoplasm, **(E)** nucleus and **(F)** mitochondria, as measured by electron microscopy. Scale bars, 500 nm **(C)** and 100 nm **(D-F)**.

**Figure 5:**
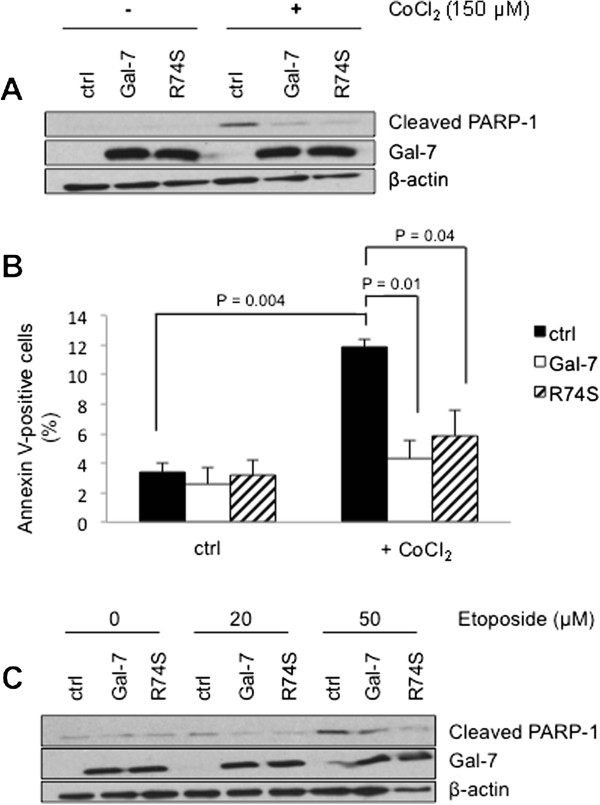
**Anti-apoptotic function of gal-7 is not altered by R74S mutation.** Effect of wild-type and mutated gal-7 on **(A)** PARP-1 cleavage and **(B)** Annexin V positive cells induced upon treatment with 150 μM CoCl_2_. **(C)** Western blot analysis of PARP-1 cleavage for increasing doses of etoposide for 24 hrs at 37°C. Controls included stable MCF-7 cells transfected with the empty (srα) expression vector.

**Figure 6:**
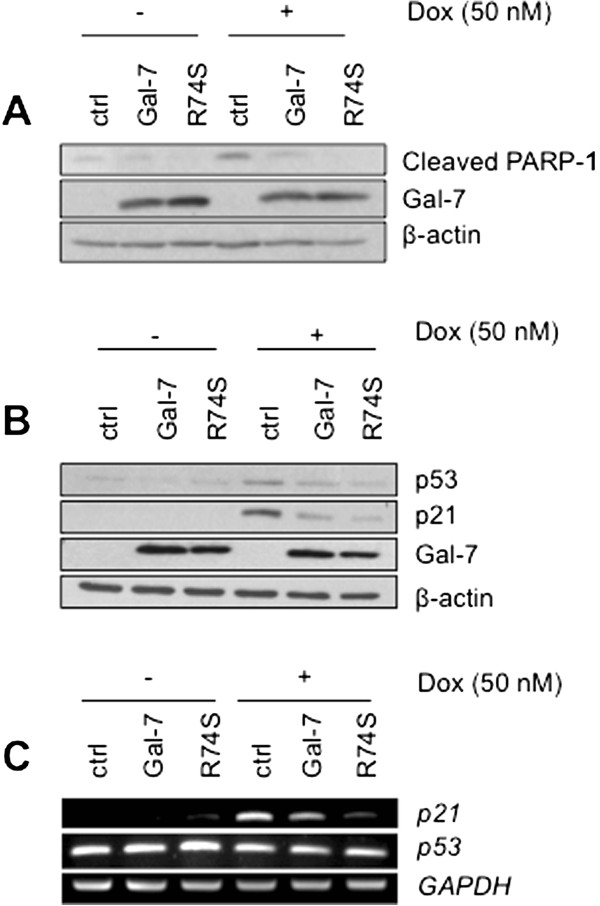
**Gal-7 and its R74S variant suppress PARP-1 cleavage, p53 expression and p21 transcription induced by doxorubicin. (A)** Stable transfectants of MCF-7 cells expressing wild type or R74S mutant gal-7 were treated with 50 nM doxorubicin for 72 hrs at 37°C and immunoblotted with cleaved PARP-1-specific antibody. **(B)** Western blot analysis showing p53, p21 and gal-7 protein expression following doxorubicin treatment for 24 hrs. **(C)** mRNA levels of *p53* and *p21* were assayed by semi-quantitative RT-PCR. β-actin and GAPDH were used as loading controls.

Because MCF-7 cells have been extensively used as a model system for human breast cancer, we have used this cell line to express and further characterize the R74S mutant. The relevance of MCF-7 model for our studies was first established by expressing the wild-type form of gal-7 (wtgal-7). Western blotting analysis of stable transfectants showed that wtgal-7 was present in cytosolic, nuclear, and mitochondrial extracts of MCF-7 cells transfected with an expression vector encoding wtgal-7 (Figure 3A-B), a pattern similar to that found in MDA-MB-468 cells. Immunogold immunohistochemistry by electron microscopy (EM) confirmed the presence of gal-7 in these subcellular compartments (Figure 3C-F). The gold beads labeled mitochondria on the outer membrane and inside the organelle. In all cases, gal-7 was expressed in clusters, mostly being found inside the mitochondria (Additional file 3: Figure S3). MCF-7 expressing wtgal-7 was also more resistant to apoptosis induced by doxorubicin (dox) as compared to control MCF-7 cells transfected with a (empty) control vector (Figure 3G). These results corroborated our previous data using the mouse 4T1 breast cancer cells [6]. We thus examined whether the R → S mutation at position 74 induced a change in the subcellular localization of gal-7. Our Western blot analyses showed that this mutation greatly reduced the expression of gal-7 in the nucleus and mitochondria (Figure 4A-B). This effect was confirmed by EM studies (Figure 4C-F). Interestingly, however, we found that MCF-7 cells expressing wild-type and R74S forms of gal-7 were equally resistant to apoptosis induced by cobalt chloride, a hypoxia mimicking agent (Figure 5A-B), and by other anticancer drugs, such as etoposide and doxorubicin (dox) (Figures 5C and 6A). In fact, MCF-7 cells expressing R74S were more resistant to apoptosis induced by etoposide and dox.

### Gal-7 reduces p53-induced p21 expression

**Figure 7:**
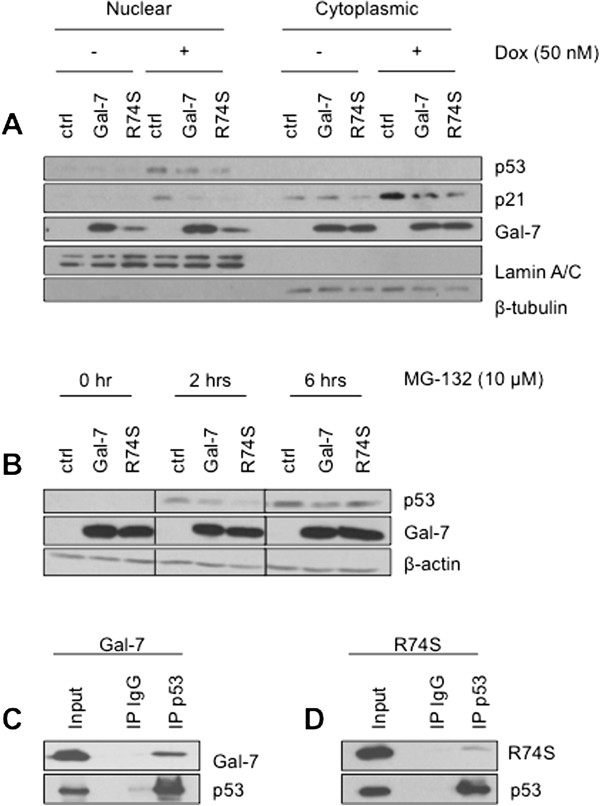
**Decreased of p53 nuclear translocation through proteasomal degradation induced by cytoplasmic gal-7. (A)** Cytosolic and nuclear fractions were purified from control (srα) cells and MCF-7 cells expressing wild type or mutated gal-7 after doxorubicin treatment. Expression of p53, p21 and gal-7 was measured in both fractions by Western blotting. β-tubulin and lamin A/C are shown as positive cytoplasmic and nuclear expression controls, respectively. **(B)** Cells were treated with 10 μM MG-132 for 0, 2, and 6 hrs. Total cellular extracts were subjected to Western blot analysis for p53, gal-7 and β-actin. Immunoprecipitation (IP) experiments showing p53 interaction with **(C)** wild type and **(D)** R74S mutant of gal-7 in MCF-7 cells. Whole-cell lysates were made from MCF-7 cells transfected with a construct expressing p53. Lysates were immunoprecipitated with anti-p53 or a control IgG antibody, and immunoblot analysis was performed with anti-gal-7.

Because MCF-7 cells express a wild-type form of p53 and that DNA damage response induced by dox is known to increase the cyclin-independent kinase inhibitor p21 via a p53-dependent pathway, we took this opportunity to examine the expression of p21 and p53 in MCF-7 cells expressing wtgal-7 and R74S. We found that both wtgal-7 and R74S inhibited dox-induced PARP-1 cleavage and p53 protein expression (Figure 6A-B). The inhibition of p53 correlated with a decrease in p21 protein expression and *CDKN1A* mRNA (Figure 6B-C). Again, the inhibition by R74S was stronger than that observed with the wild-type form of gal-7. Furthermore, analysis of nuclear and cytoplasmic fractions showed that wild type and R74S mutant gal-7 inhibited p53 nuclear translocation (Figure 7A). Treatment of cells with MG-132, a well-known proteasome inhibitor, restored the p53 expression, suggesting that both forms of gal-7 promote degradation of cytosolic p53 (Figure 7B). This possibility is supported by our data showing that both forms of gal-7 co-precipitate with p53 (Figure 7C-D). The ability of endogenous gal-7 to co-precipitate with p53 was further confirmed using MCF10A cells (Additional file 4: Figure S4).

## Discussion

There is an increasing interest in the development of galectin-specific inhibitors for the treatment of cancer. Because galectins exert both intracellular and extracellular functions, a better understanding of their subcellular localization in cancer cells is critical to promote the development of new anti-cancer therapies directed at these proteins.

Gal-7 is highly expressed at both the mRNA and protein levels in tissues of patients with aggressive forms of cancer, including basal-like breast cancer subtype [6, 19, 20]. Experimentally, gal-7 has been shown to increase the metastatic behavior of cancer cells while its suppression reduces their metastatic behavior [6, 21, 22]. Like other members of the galectin family, however, gal-7 has been shown to have a dual role in cancer. While it promotes cancer progression in many types of cancer, it may exert an anti-tumor activity in other types of cancer, such as urothelial carcinoma and colon cancer. In all cases, the role of gal-7 in apoptosis was associated with its intracellular localization, as shown by the strong cytosolic and nuclear immunoreactivity with anti-gal-7 antibodies [6, 23]. Our model system with the R74S mutant will thus be useful to determine whether translocation to mitochondria and nucleus modulates the ability of gal-7 to modulate apoptosis in other cancer cell types.

Because R74 is located in the vicinity of the CRD, it is not surprising that a mutation at this position reduces the affinity to lactose or the binding to cell surface glycoproteins. It may also affect the fine specificities of ligand recognition in the ligand binding groove, as suggested by the tridimensional structure of gal-7 [4]. We expect, however, that the R74S mutation will not affect the protein-protein interactions that gal-7 displays with proteins such as Bcl-2 and Smad3 [9, 11]. Similarly to galectin-3 that utilizes synexin for its translocation to the perinuclear mitochondrial membranes, gal-7 might also require the aid of similar transport proteins for its translocation [24]. As such, the cytosolic presence of the R74S mutant is potentially due to the loss of the interaction between gal-7 and its transport proteins resulting in its pronounced cytosolic localization. Alternatively, the mutation at the R74 promoting the cytosolic sequestration of gal-7 may allow enhanced binding to cytosolic proteins increasing as such various cytoplasmic signaling pathways. Nevertheless, future studies will be needed to determine the specific mechanism by which gal-7 translocates to mitochondria and to the nucleus.

Our results suggest that gal-7 may be involved in the regulation of p21 expression. These results may thus provide a new mechanism underlying the functions of gal-7 in apoptosis and warrant further investigation. Specifically, we found that the R74S mutation does not alter the proliferation rate of breast cancer cells (Additional file 5: Figure S5). Rather, our data obtained using the proteasome inhibitor and the co-immunoprecipitation of gal-7 with p53 suggests that gal-7 may help to stabilize cytosolic p53, possibly by modulating its interaction with MDM2. Whether gal-7 directly binds to p53 or belongs to the p53 multimolecular complex is currently unknown. Although glycosylation of p53 has been reported [25–27] and that some p53-interacting proteins are glycosylated [28], our observation that R74S also co-immunoprecipitates with p53 suggests that such interaction could be CRD-independent. Such CRD-independent function for galectins is not uncommon, especially for intracellular galectins [3]. Another possibility that may explain lower levels of nuclear p53 protein and reduced p21 activation is that gal-7 may be part of a complex network of interrelated mechanisms that regulate the nucleo-cytoplasmic transport of p53 following cellular stress. These possibilities are currently under investigation.

## Conclusions

In the present work, we have shown that: 1) a mutation at position 74 inhibited translocation of gal-7 to the mitochondria and the nucleus, sequestering gal-7 to the cytosolic compartment; 2) such decrease of gal-7 expression in the nucleus and mitochondria does not impair the ability of gal-7 to drug-induced apoptosis; in fact, the R74S mutant protected even more cells from apoptosis induced by anti-cancer drugs, and 3) sequestration of gal-7 to the cytosol impaired the translocation of p53 to the nucleus and the upregulation of p21. Taken together, these results suggest that targeting cytosolic gal-7 in breast cancer cells may be a valuable strategy for the treatment of this disease.

## Electronic supplementary material

Additional file 1: Figure S1: Isothermal calorimetric titration of gal-7 with lactose. (Top) Typical ITC experiment carried out by adding 2 μl aliquots of 6 mM lactose to 200 μM wtgal-7 or R74S. (Bottom) Heat released per mole of lactose injected as a function of the sugar / protein molar ratio. The titration was obtained at 25°C in 20 mM potassium phosphate buffer, pH 7.2. (PNG 135 KB)

Additional file 2: Figure S2: Binding of recombinant wtgal-7 and R74S to Jurkat T cells. Increasing concentrations of recombinant FITC-labeled wtgal-7 or R74S were added to Jurkat T cells. Binding was measured by flow cytometry after 30 min of incubation at 4°C. (PNG 79 KB)

Additional file 3: Figure S3: Clusters of gal-7 in MCF-7 cells observed by electron microscopy. Transmission electron micrographs showing gal-7 clusters (arrows) in (A) the cytoplasm, (B) nucleus, and (C) mitochondria of MCF-7 stable transfectants expressing gal-7. In (D), quantitative assessment of the number of immunogold particles per clusters as measured from transmission electron micrographs. (PNG 272 KB)

Additional file 4: Figure S4: Interaction of endogenous gal-7 with p53 in MCF10A cells. Immunoprecipitation (IP) experiments showing p53 interaction with endogenous gal-7 in MCF10A cells. Whole-cell lysates were made from MCF10A cells transfected with a construct expressing p53. Lysates were IP with anti-p53 or a control IgG antibody, and immunoblot analysis was performed with anti-gal-7. (PNG 68 KB)

Additional file 5: Figure S5: Cellular proliferation of MCF-7 is not significantly affected by gal-7 and R74S variant. Stable transfectants of MCF-7 cells expressing empty vector (ctrl), wild type or R74S mutant gal-7 were seeded at 5000 cells per well in E-Plates 96 and observed for a period of 72 h. Dynamic proliferation assay was achieved via xCELLigence real-time cell analyzer. (PNG 122 KB)

## Abbreviations

gal-7: Galectin-7
wtgal-7: Wild-type galectin-7
CRD: Carbohydrate recognition domain
dox: Doxorubicin.
