# Supplementary figures and images for: Cytosolic galectin-7 impairs p53 functions and induces chemoresistance in breast cancer cells
Source: BMC Cancer. 2014 Nov 3;14:801. doi: 10.1186/1471-2407-14-801 (PMC4228062; doi:10.1186/1471-2407-14-801)

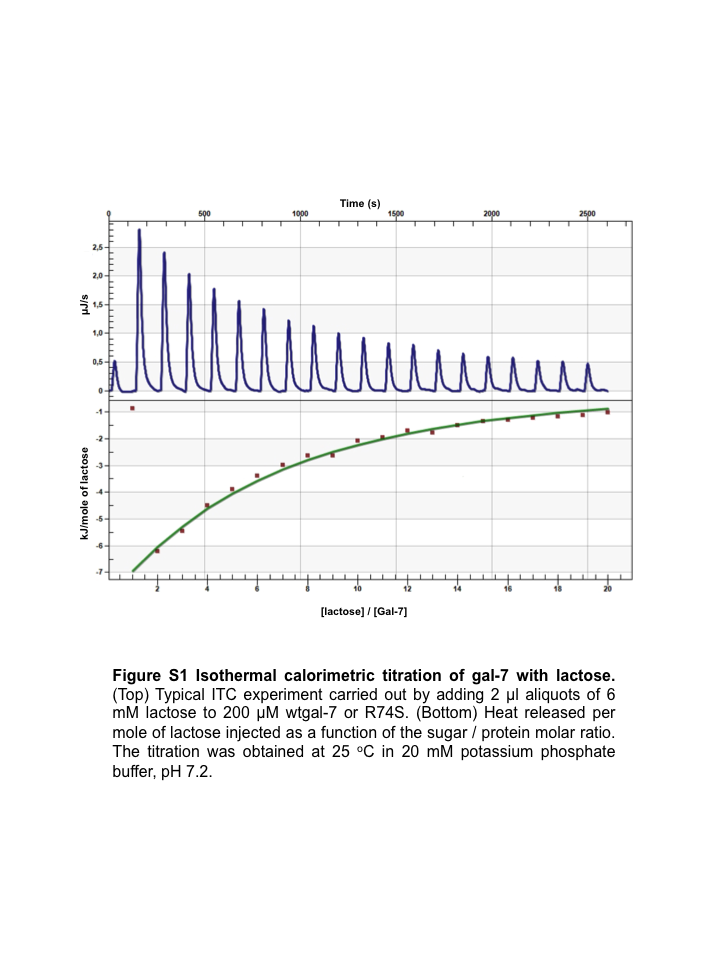

Supplement: Supplementary file 1 — Additional file 1: Figure S1: Isothermal calorimetric titration of gal-7 with lactose. (Top) Typical ITC experiment carried out by adding 2 μl aliquots of 6 mM lactose to 200 μM wtgal-7 or R74S. (Bottom) Heat released per mole of lactose injected as a function of the sugar / protein molar ratio. The titration was obtained at 25°C in 20 mM potassium phosphate buffer, pH 7.2. (PNG 135 KB) [file 12885_2014_4975_MOESM1_ESM.png]

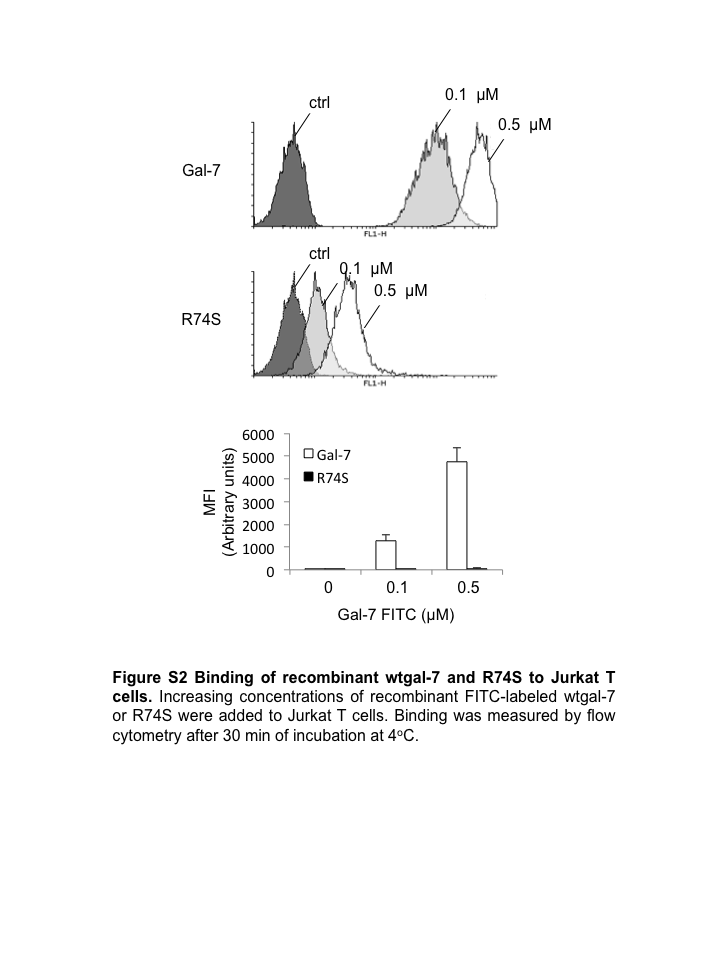

Supplement: Supplementary file 2 — Additional file 2: Figure S2: Binding of recombinant wtgal-7 and R74S to Jurkat T cells. Increasing concentrations of recombinant FITC-labeled wtgal-7 or R74S were added to Jurkat T cells. Binding was measured by flow cytometry after 30 min of incubation at 4°C. (PNG 79 KB) [file 12885_2014_4975_MOESM2_ESM.png]

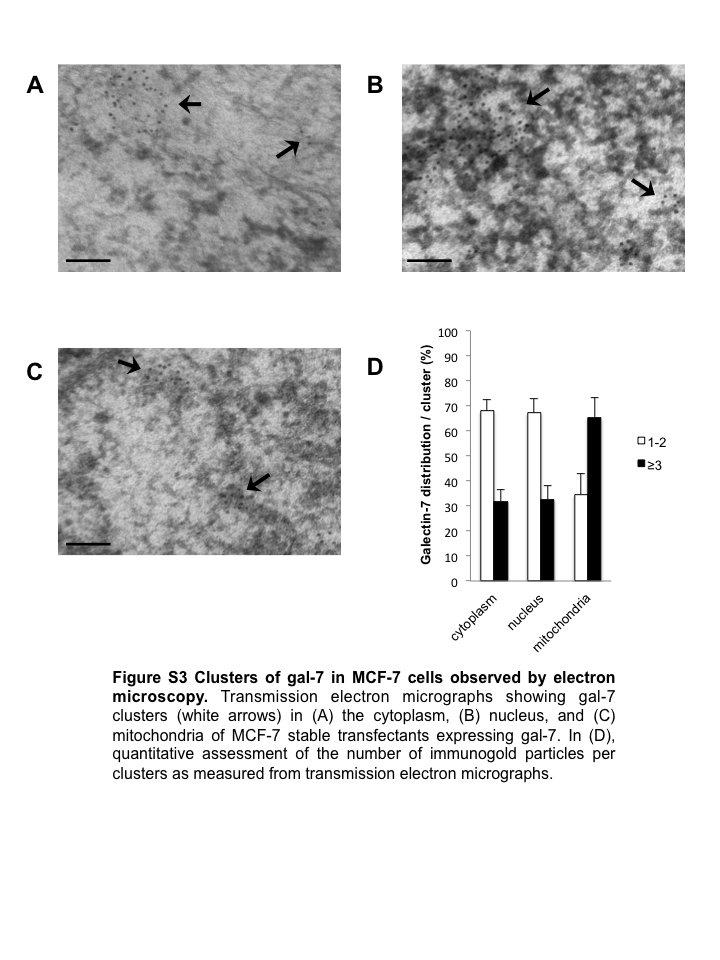

Supplement: Supplementary file 3 — Additional file 3: Figure S3: Clusters of gal-7 in MCF-7 cells observed by electron microscopy. Transmission electron micrographs showing gal-7 clusters (arrows) in (A) the cytoplasm, (B) nucleus, and (C) mitochondria of MCF-7 stable transfectants expressing gal-7. In (D), quantitative assessment of the number of immunogold particles per clusters as measured from transmission electron micrographs. (PNG 272 KB) [file 12885_2014_4975_MOESM3_ESM.png]

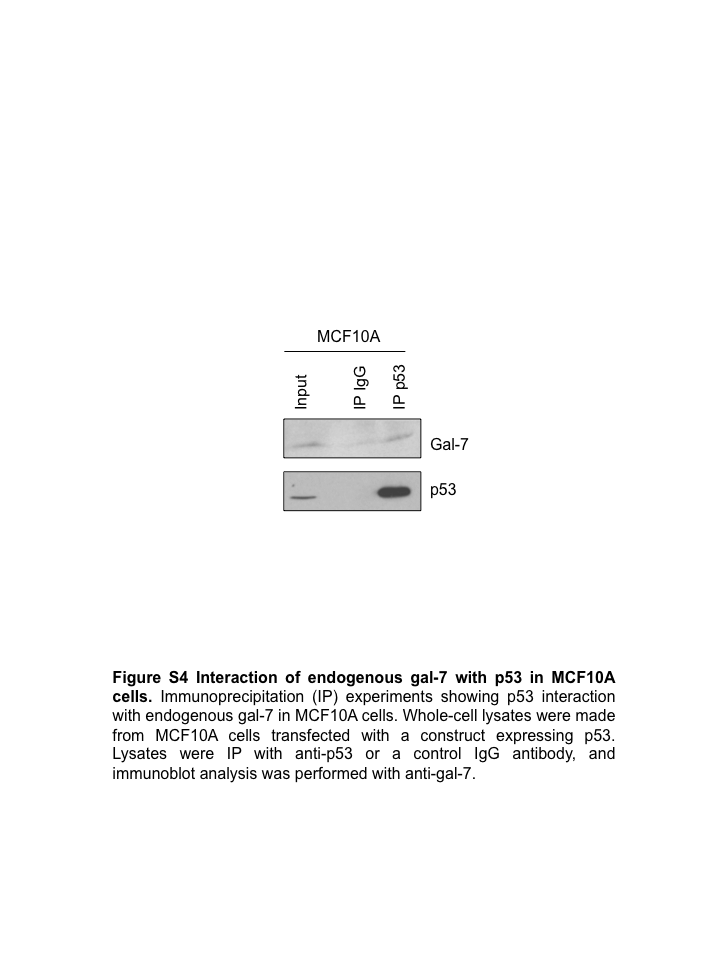

Supplement: Supplementary file 4 — Additional file 4: Figure S4: Interaction of endogenous gal-7 with p53 in MCF10A cells. Immunoprecipitation (IP) experiments showing p53 interaction with endogenous gal-7 in MCF10A cells. Whole-cell lysates were made from MCF10A cells transfected with a construct expressing p53. Lysates were IP with anti-p53 or a control IgG antibody, and immunoblot analysis was performed with anti-gal-7. (PNG 68 KB) [file 12885_2014_4975_MOESM4_ESM.png]

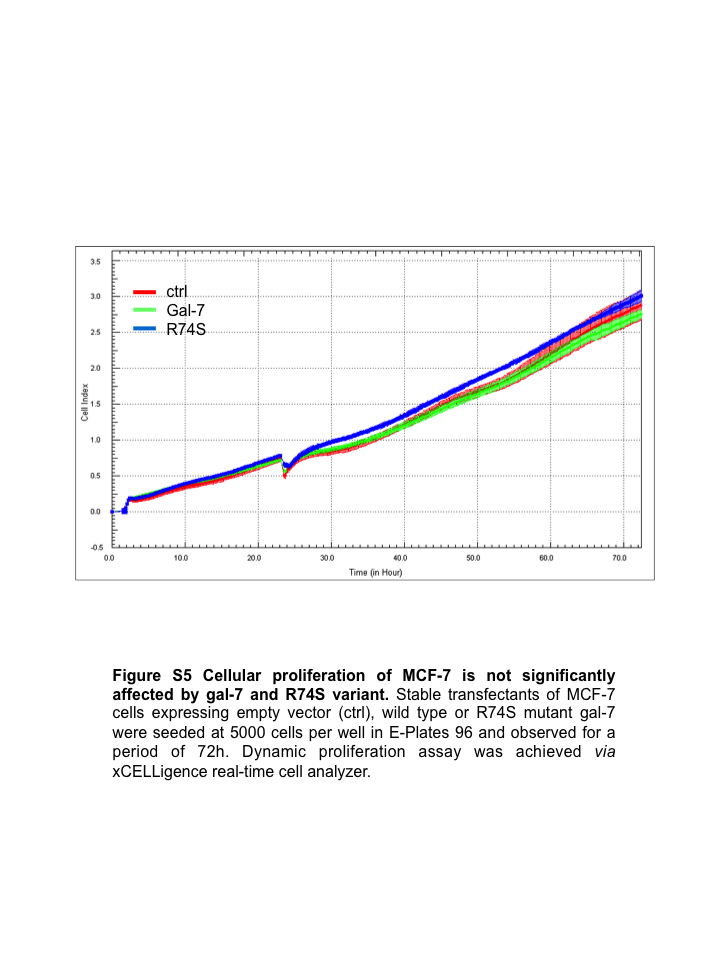

Supplement: Supplementary file 5 — Additional file 5: Figure S5: Cellular proliferation of MCF-7 is not significantly affected by gal-7 and R74S variant. Stable transfectants of MCF-7 cells expressing empty vector (ctrl), wild type or R74S mutant gal-7 were seeded at 5000 cells per well in E-Plates 96 and observed for a period of 72 h. Dynamic proliferation assay was achieved via xCELLigence real-time cell analyzer. (PNG 122 KB) [file 12885_2014_4975_MOESM5_ESM.png]
